# VEGF induces sensory and motor peripheral plasticity, alters bladder function, and promotes visceral sensitivity

**DOI:** 10.1186/1472-6793-12-15

**Published:** 2012-12-19

**Authors:** Anna P Malykhina, Qi Lei, Chris S Erickson, Miles L Epstein, Marcia R Saban, Carole A Davis, Ricardo Saban

**Affiliations:** 1 Department of Surgery, Division of Urology, University of Pennsylvania School of Medicine, Glenolden, PA 19036-2307, USA; 2 Department of Neurosciences, University of Wisconsin-Madison, Madison, WI 53706, USA; 3 Department of Physiology, College of Medicine, Urinary Tract Physiological Genomics Laboratory, University of Oklahoma Health Sciences Center (OUHSC), 800 Research Parkway, Room 410, Oklahoma City, OK 73104, USA

## Abstract

**Background:**

This work tests the hypothesis that bladder instillation with vascular endothelial growth factor (VEGF) modulates sensory and motor nerve plasticity, and, consequently, bladder function and visceral sensitivity.

In addition to C57BL/6J, ChAT-cre mice were used for visualization of bladder cholinergic nerves. The direct effect of VEGF on the density of sensory nerves expressing the transient receptor potential vanilloid subfamily 1 (TRPV1) and cholinergic nerves (ChAT) was studied one week after one or two intravesical instillations of the growth factor.

To study the effects of VEGF on bladder function, mice were intravesically instilled with VEGF and urodynamic evaluation was assessed. VEGF-induced alteration in bladder dorsal root ganglion (DRG) neurons was performed on retrogradly labeled urinary bladder afferents by patch-clamp recording of voltage gated Na+ currents. Determination of VEGF-induced changes in sensitivity to abdominal mechanostimulation was performed by application of von Frey filaments.

**Results:**

In addition to an overwhelming increase in TRPV1 immunoreactivity, VEGF instillation resulted in an increase in ChAT-directed expression of a fluorescent protein in several layers of the urinary bladder. Intravesical VEGF caused a profound change in the function of the urinary bladder: acute VEGF (1 week post VEGF treatment) reduced micturition pressure and longer treatment (2 weeks post-VEGF instillation) caused a substantial reduction in inter-micturition interval. In addition, intravesical VEGF resulted in an up-regulation of voltage gated Na^+ ^channels (VGSC) in bladder DRG neurons and enhanced abdominal sensitivity to mechanical stimulation.

**Conclusions:**

For the first time, evidence is presented indicating that VEGF instillation into the mouse bladder promotes a significant increase in peripheral nerve density together with alterations in bladder function and visceral sensitivity. The VEGF pathway is being proposed as a key modulator of neural plasticity in the pelvis and enhanced VEGF content may be associated with visceral hyperalgesia, abdominal discomfort, and/or pelvic pain.

## Background

It is highly likely that neurogenic dysfunction of the urinary bladder is involved in various disorders of the lower urinary tract (LUT) including neurogenic bladder, outflow obstruction, idiopathic detrusor instability, overactive bladder, painful bladder syndrome, and diabetic neuropathy. In addition, chronic pathological conditions that cause tissue irritation or inflammation can alter the properties of sensory pathways, leading to a reduction in pain threshold and/or an amplification of painful sensation (hyperalgesia) [1].

Depending on the pathology, several mediators and their respective receptors have been proposed to modulate peripheral nerve plasticity in the LUT, including but not limited to: purinergic receptors in general [2] or P2X receptor in particular [3], TRPV1 [1,4], substance P acting on NK1 receptors [5], protease activated receptors [6], and nerve growth factor and its receptors [7].

In this context, the development of cross-sensitization in the pelvis is one of the suggested mechanisms underlying co-morbidity of pelvic disorders which is frequently observed in the clinical setting [8]. Recently, evidence indicated that acute colonic inflammation triggers the occurrence of urinary bladder detrusor instability via activation of the transient receptor potential vanilloid subfamily 1 (TRPV1) related pathways [4]. Moreover, colonic inflammation-induced activation of TRPV1 receptors at the peripheral sensory terminals results in an up-regulation of voltage gated Na^+^ channels on the cell soma of bladder sensory neurons [9]. This increase in channels may underlie the occurrence of peripheral cross-sensitization in the pelvis and functional chronic pelvic pain [9].

The new hypothesis being tested in this manuscript is that increased levels of VEGF observed during bladder inflammation provoke nerve plasticity. This hypothesis is based on evidence indicating that nerves and blood vessels are anatomically associated, follow a common molecular pathway during development, and their maturation in adulthood may be controlled by the same key molecules responsible for their development [10,11]. The finding that mutant mice (neurogenin1/neurogenin2 double knockout embryos) lacking sensory nerves also show disorganized blood vessel branching [12], suggests that local signals such as VEGF supplied by nerve fibers, may provide a cue that determines blood vessel patterning.

Evidence has been presented supporting the hypothesis that many proteins that were originally discovered to be required for axon guidance are implicated in the development of the vascular [11] and lymphatic systems [13]. But perhaps the most striking observation linking the nervous and vascular systems is the finding that angiogenic factors, when deregulated, contribute to various neurological disorders, such as neurodegeneration. The prototypic example of this cross-talk between nerves and vessels is the vascular endothelial growth factor, VEGF [14]. Although originally described as a key angiogenic and permeability factor, it is now well established that VEGF also plays a crucial role in the development of the nervous system [14].

Recently, we provided evidence that chronic inflammation increases the density of bladder sensory nerves that express: a) the transient receptor potential vanilloid subfamily 1 (TRPV1) [15], b) protein gene product (PGP9.5) [16], c) substance P, and d) calcitonin gene-related peptide (CGRP) [17]. We also determined that B20, a VEGF neutralizing antibody, prevented inflammation-induced increase in sensory nerves [17]. Furthermore, instillation of VEGF into the bladder recapitulated the effect of inflammation on sensory nerve plasticity [17], and represents direct evidence of VEGF action on the peripheral nervous system.

The scope of the present work was to determine whether VEGF, in addition to increased sensory nerve density, also alters the density of cholinergic nerves, and, consequently, bladder function and visceral sensitivity.

## Results

### Instillation of VEGF into the mouse bladder results in an increase in sensory nerve density

**Figure 1:**
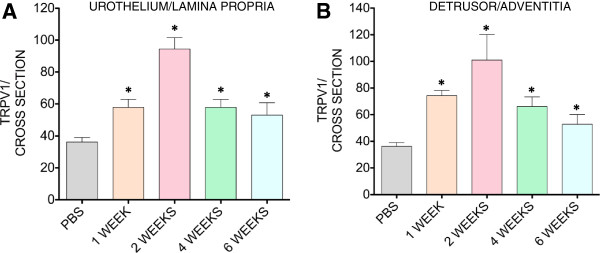
**Intravesical instillation of VEGF induces a time-dependent alteration in the density of TRPV1-positive sensory fibers in the urothelium and lamina propria (A) and detrusor and and adventitia (B) layers.** Quantification of TRPV1-IR in the C57BL/6 urinary bladder isolated 1 week after: 1, 2, 4, and 6 weekly instillations of VEGF of PBS (N=8 per group). Asterisks indicate a statistically significant difference (*p* values < 0.05) when compared to the PBS group.

It was reported that VEGF is expressed at relatively higher amounts in nerves than in the surrounding mesenchymal tissue [12]. This finding led to a new appreciation of the role of VEGF in neuronal development [14,18] and stimulated us to review a possible link between VEGF-induced inflammation and bladder nerve plasticity. To provide direct evidence that VEGF induces bladder neuronal plasticity, VEGF was instilled into the C57BL/6 mouse bladder. Previous results from our laboratory indicated that acute or chronic instillation of VEGF into the mouse bladder caused inflammation, characterized predominantly by the accumulation of macrophages [17], and an increase in sensory nerve density as indicated by image analysis of nerve fibers positive for the nociceptive transducer vanilloid type 1 transient receptor potential receptor (TRPV1) [17]. In the present manuscript, we expanded the time course of VEGF exposure by including a group that received two weekly instillations of VEGF. Female C57BL/6 mice were instilled weekly for two weeks with VEGF (6.41 nM in 100 μl). Mice were euthanized one week after the second VEGF instillation and the bladder was removed for image analysis of TRPV1-positive fibers. One VEGF instillation promotes a substantial increase in TRPV1-positive fibers in the urothelium and lamina propria (Figure 1A) and in the detrusor and adventitia (Figure 1B). Two VEGF instillations resulted in the most pronounced alteration in sensory nerve plasticity, as indicated by a peak increase in TRPV1-positive fibers. After the 4^th ^weekly treatment with VEGF, the response was reduced. Similar results were obtained with ChAT mice indicating that 1 and 2 weekly VEGF instillations provoked similar increases in TRPV1 immune reactivity (data not shown). These results provide direct evidence that VEGF participates in the plasticity of bladder sensory nerves and that two weekly instillations of VEGF produced the largest increase in sensory nerve density.

### A mouse model for the study of bladder cholinergic innervation

**Figure 2:**
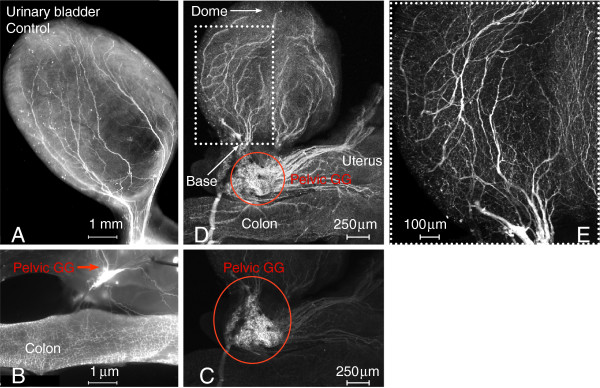
**A ChAT transgenic mouse permits the visualization of cholinergic cells and fibers in a pelvic ganglion and cholinergic fibers in the urinary bladder, gastrointestinal tract, and uterus.** Representative photomicrographs of whole mount preparations of the urinary bladder removed from ChAT mice instilled with PBS (**A**). The pelvic ganglion and colon are also illustrated. (**B**). ChAT nerves appeared intact confirming previous results that intravesical instillation does not cause damage to the bladder wall. **C**, **D**, and **E** are confocal photomicrographs of whole mount preparations isolated from another control ChAT mouse. An intense fluorescence was observed in the pelvic ganglion region (**C**). In order to permit visualization of the ChAT-positive fibers in the bladder, uterus, and colon, the intensity of the pelvic ganglion (red circle) was reduced. **E** is a high magnification of the area delimited by the white dotted rectangle in figure **D** and permits the visualization of large and small cholinergic fibers innervating the urinary bladder.

We sought to extend our studies by investigating whether VEGF also alters the plasticity of bladder cholinergic nerves. The rationale for the use of the ChAT-cre mouse model was based on reports that staining peripheric nerves with antibodies targeting the synthesizing enzyme choline acetyltransferase (ChAT) is not consistent and often fails to detect these neurons. In contrast, cholinergic neurons and fibers of the urinary bladder and surrounding tissues are readily visualized in whole mount preparations isolated from adult ChAT mice (Figure 2A and 2B). High quality photomicrographs were used to detailed the disctribution of ChAT-positive nerves (Figure 2C, 2D, and 2E). However, using these high quality micrographs, we found the pelvic ganglia to be so intensely fluorescent that the signals from the surrounding tissues were not readily apparent (Figure 2C, red circle). When the fluorescence of the PG region was electronically reduced, it was possible to image the innervation of the colon, uterus, and urinary bladder (Figure 2D). Both preparations also allow the visualization of the pelvic ganglia and its relationship with cholinergic nerves innervating the urinary bladder and other pelvic organs (Figure 2B and 2C), which will permit the capture of these cells for future patch clamp studies. Figure 2E is a high magnification image of the dotted square region of Figure 2D and shows the large superficial nerve trunks innervating the adventitial coat. As described in cross-sections presented below, these fibers penetrate deep into the lamina propria and ramify in the urothelium.

**Figure 3:**
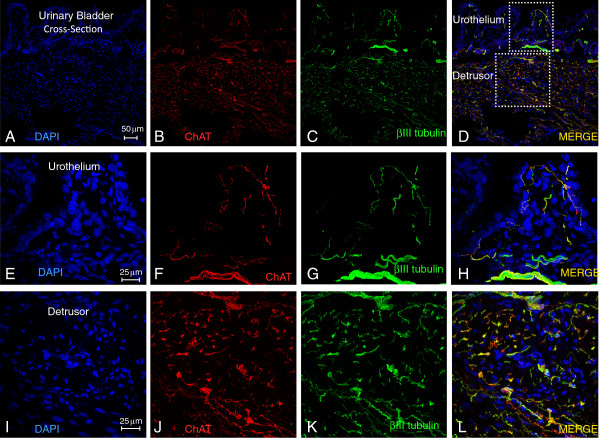
**ChAT–positive fibers are nerve elements.** Representative photomicrographs of 12 μm thick cross-sections of the urinary bladder isolated from ChAT mice. Sections were stained with DAPI (4',6-diamidino-2-phenylindole) to highlight the cell nucleus and with class III β-tubulin antibody. **A**-**D** illustrates that both β-III tubulin- and ChAT-positive fibers are distributed within the urothelium and detrusor smooth muscle. Areas highlighted by dotted squares in **D** were photographed at higher magnification to show the overlap of beta-III tubulin(green)- and ChAT(red)- positive fibers fluorescence in the urothelium (**E**-**H**) and detrusor smooth muscle (**I**-**L**).

In order to confirm that ChAT positive fibers crossing the urinary bladder were indeed nerve fibers, we stained with an antibody targeting the class III β-tubulin, a specific neuronal marker [19]. Both β-III tubulin- and ChAT-positive fibers are distributed within the urothelium and detrusor smooth muscle (Figure 3A-D). Areas highlighted by dotted squares in Figure 3D were photographed at higher magnification to discriminate the overlap of the green and red fluorescence in the urothelium (Figure 3E-H) and detrusor smooth muscle (Figure 3I-L). These images indicate substantial overlap between the cholinergic and tubulin markers and thus show that the cholinergic fibers are indeed neuronal.

**Figure 4:**
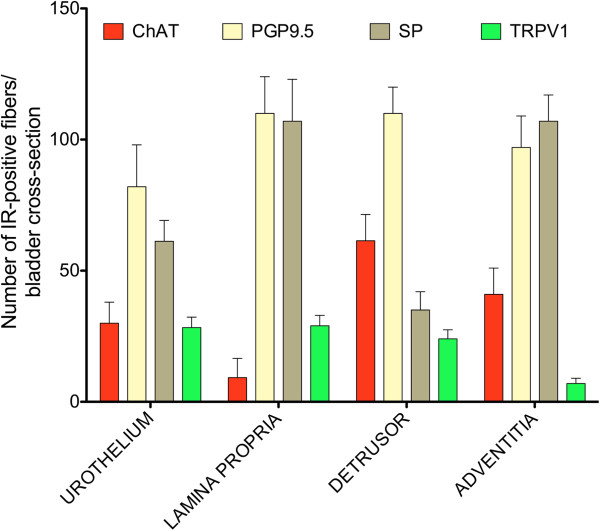
**Relative density of ChAT-positive nerve fibers in comparison with PGP9.5, Substance P (SP), and TRPV1.** Mouse urinary bladders were isolated from control ChAT mice and stained for the pan neuronal marker PGP9.5, TRPV1, and SP. The density of immunoreactive fibers was counted in 6-10 non-overlapping cross-sectional images of the urinary bladder (400X magnification). The number of fibers (length between 0.19-500 μm and width 0.19-2.5 μm) stained positively by a particular antibody per cross-sectional area was calculated and the results of 6-10 fields were averaged. This procedure was repeated for 5 bladders and the results are expressed as mean ± SEM.

An analysis of images from sections shows the relative density of ChAT fluorescent fibers in the urothelium, lamina propria (sub-urothelium), detrusor muscle, and adventitia in comparison with areas immunostained by a pan-neuronal marker (PGP9.5), sensory nerves (TRPV1), and substance P containing fibers (Figure 4). It has to be noted that PGP9.5 antibodies resulted in a non-specific labeling of the urothelial layer. A reasonable explanation for this artifact is that the methods recommended for permeabilization of the tissues caused such labeling. These findings precluded the use of the urothelium for quantification purposes of PGP9.5 but not for TRPV1 and SP. PGP9.5 image analysis was performed in two layers: the detrusor smooth muscle and the sub-urothelium that extended from the basal layer of the urothelium to detrusor. Overall, these results indicate ChAT nerves are more prominent in the detrusor muscle when compared to TRPV1 nerves, whereas, TRPV1 nerves are more prominent than ChAT nerves in the lamina propria.

**Figure 5:**
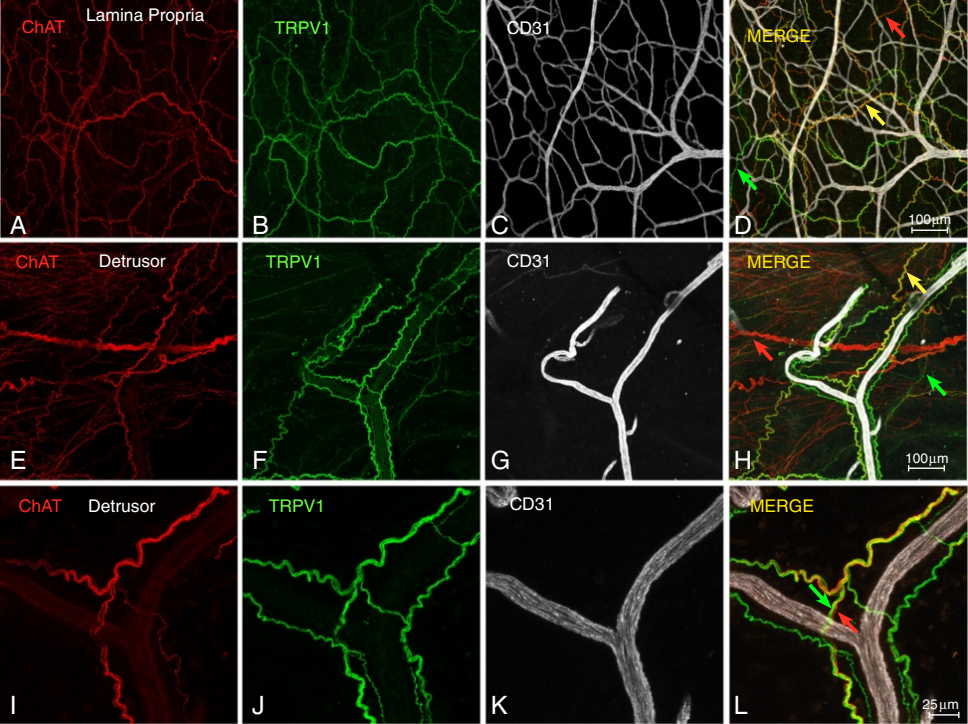
**Representative photomicrographs of bladder whole mounts showing ChAT-positive nerve fibers, sensory nerves (TRPV1), and blood vessels (CD31).** Representative photomicrographs taken from bladders isolated from ChAT mice and prepared as whole mounts that underwent blunt dissection to separate the lamina propria from the detrusor. In the lamina propria (**D**) the ChAT and TRPV1 fibers are usually associated (yellow arrow) but sometimes take separate paths (red and green arrows). In the detrusor (**H**) ChAt-positive fibers dominate although the ChAT and TRPV1 fibers around blood vessels are usually associated (yellow arrow). High magnification microphotographs (**I**-**L**) indicate that the two type of fibers run adjacent to each other (**L**).

**Figure 6:**
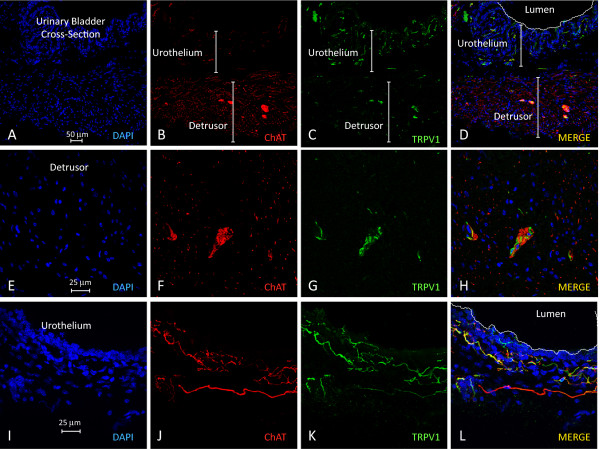
**Photomicrographs of bladder cross-sections confirm the results obtained with whole mounts and indicate the predominance of TRPV1 fibers in the urothelium and the predominance of ChAT fibers in the detrusor smooth muscle. A**-**D** are representative photomicrographs of the entire bladder cross section. **E**-**H** are representative photomicrographs of the detrusor smooth muscle taken at high magnification to show some areas of overlap between ChAT and TRPV1, primarily in intramural fascicles (**H**). **I**- **L** are representative photomicrographs of bladder urothelium taken at high magnification to show some areas of overlap between ChAT and TRPV1, primarily in the lamina propria (sub urothelium), as illustrate (**L**).

The finding that both sensory (TRPV1-positive) and cholinergic nerves are involved in urinary tract disorders such as overactive bladders [20] raises the question of whether these two systems are anatomically distinct. Therefore, we sought to determine whether sensory and motor nerves are co-localized in urinary bladder. Using bladder whole mounts that underwent blunt dissection to separate the lamina propria from the detrusor (Figure 5), we observed that TRPV1 and ChAT signals appear to overlap in the lamina propria (yellow arrow on Figure 5D) and to a lesser degree in the detrusor smooth muscle (Figure 5H). However, high magnification photomicrographs (Figures 5I-L) indicate that the two types of fibers are separate and run adjacent to each other, primarily around the blood vessels (Figure 5L). Photomicrographs of bladder cross-sections confirm the results obtained with whole mounts (Figure 6D). In addition, higher magnification images illustrate the separation of TRPV1 and ChAT fibers in intramural ganglia (Figure 6H) as well as in the lamina propria (Figure 6L). We conclude that the sensory and motor components do not overlap.

### VEGF instillation increases the number of ChAT positive fibers

**Figure 7:**
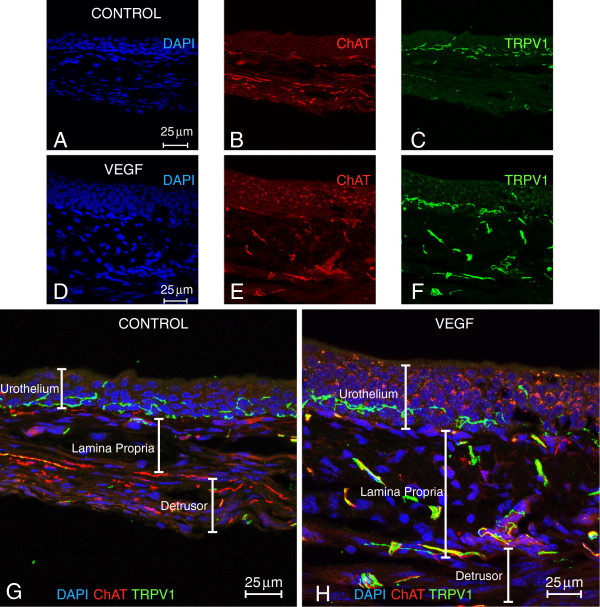
**Representative photomicrographs of ChAT mouse bladder cross-sections isolated from control and a single VEGF instillation (Table **1**).** Flattened images from 10 μm thick cross-sections from control and VEGF treated are presented side by side to permit comparison. DAPI staining highlighted the cell nuclei (**A** and **D**), ChAT was visualized by the fluorescent protein (td tomato; **B** and **E**), and sensory nerves were identified by TRPV1 immunoreactivity (**C** and **F**). Enlarged and merged microphotographs are presented to permit a better appreciation of the differences between control (**G**) and VEGF-treated bladders (**H**).

**Figure 8:**
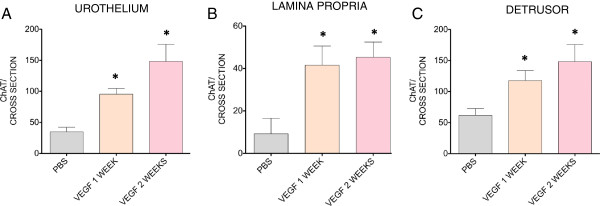
**VEGF increases the density of cholinergic nerves.** ChAT-positive fiber density in the urinary bladder isolated from ChAT mice 1 week after treatment with PBS, or 1 and 2 weeks of after VEGF instillations. The results indicate a significant increase in ChAT density in the urothelium (**A**), lamina propria (**B**), and detrusor smooth muscle (**C**). The density of immunoreactive fibers was counted in 6-10 non-overlapping cross-sectional images of the urinary bladder (400X magnification). The number of fibers (length between 0.19-500 μm and width 0.19-2.5 μm) stained positively by a particular antibody per cross-sectional area was calculated and the results of 6-10 fields were averaged. This procedure was repeated for all bladders and the results are expressed as mean ± SEM. (N=5 per group).

Figure 7 contains representative photomicrographs of urinary bladders isolated from control and VEGF-treated ChAT mice. These cross-sections suggest that VEGF treatment resulted in an increased density of ChAT- (Figure 7B and 7E) and TRPV1- positive fibers (Figure 7C and 7F). For better appreciation of sensory and cholinergic nerves, merged microphotographs were digitally amplified and are presented on Figure 7G and 7H (note the calibration bar on Figures 7G and H and compare with the bars on Figures 7A and D). Image analysis of nerve density indicates that single or repeated challenge of mouse bladders with VEGF resulted in a significant increase in ChAT density in the urothelium, lamina propria, and detrusor smooth muscle (Figure 8). Together these results indicate that in addition to alterations of peripheral sensory nerve plasticity (Figure 1), direct administration of VEGF also promotes an increase in bladder cholinergic nerve plasticity.

### Urodynamic analysis of bladder function after intravesical VEGF instillation in awake (unrestrained) mice

**Figure 9:**
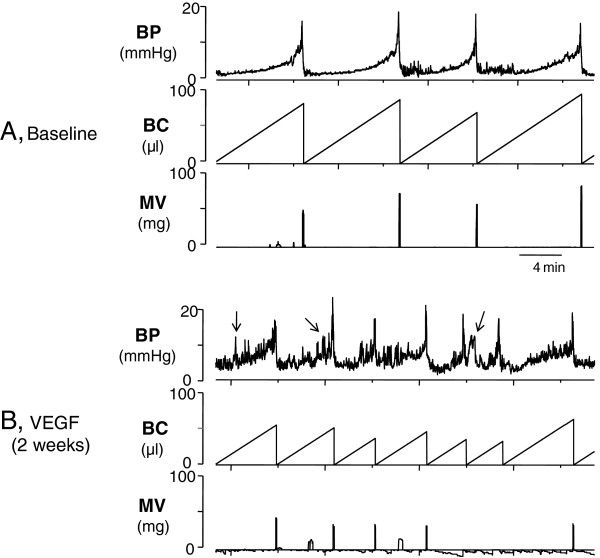
**Representative cystograms recorded in awake and freely moving mice.** Top panel shows raw traces recorded in one of the mice before (**A** = baseline) and after (**B** = lower panel) intravesical VEGF instillations. An increase in voiding frequency and the number of non-micturition contractions were observed in all mice from the VEGF group. Arrows point at non-micturition contractions after VEGF treatment. BP- Bladder pressure, BC – bladder capacity, MV – micturition volume.

To evaluate the effects of intravesical VEGF on urodynamic parameters and function of the urinary bladder *in vivo*, we performed cystometric assessment in conscious mice. Micturition cycles were first recorded under control conditions (N=5) and served as a baseline (internal control) followed by urodynamic evaluation at 1 and 2 weeks after beginning VEGF treatment. Figure 9 shows raw cystometric traces recorded in the same animal before (Figure 9A, baseline) and 2 weeks after intravesical VEGF (Figure 9B). Urodynamic parameters were first compared to the baseline for each mouse followed by further comparisons between the groups (before and after treatment). Intravesical VEGF caused significant changes in the function of the urinary bladder over the course of treatment (Figure 9B).

**Figure 10:**
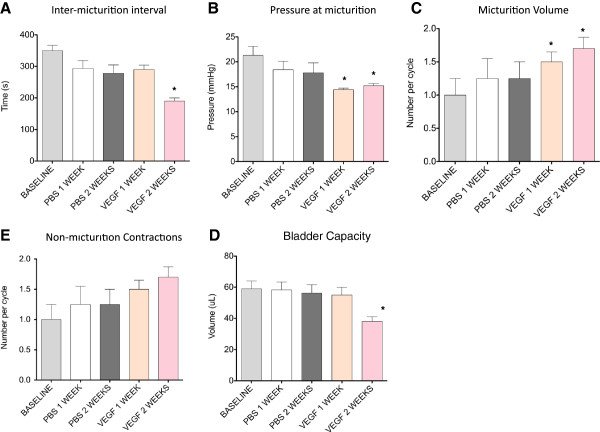
**Analysis of urodynamic parameters recorded in conscious and unrestrained mice.** Comparison of the intermicturition interval (**A**), pressure at micturition (**B**), micturition volume (**C**), bladder capacity (**D**) and number of non-voiding contractions (**E**) in mice before, after 1 and 2 weeks of intravesical vehicle instillation (PBS), and after 1 and 2 weeks of VEGF instillation. * - p≤0.05 in comparison to baseline values.

At 1 week post-VEGF, significant changes included a reduction in micturition pressure (19.75 ± 0.53 mmHg at baseline *vs* 14.4 ± 0.32 mmHg at 1 week, (Figure 10B, p≤0.005) and decreased micturition volume (by 26% in comparison to baseline, (p≤0.001, Figure 10C). These parameters were also significantly reduced 2 weeks after the initiation of VEGF treatment. Longer treatment (2 weeks) caused a substantial reduction in inter-micturition interval from 341.2 ± 25.6 s to 188.0 ± 20.8 s (p≤0.001, Figure 10A) and a decrease in bladder capacity from 57.1 ± 4.3 μl to 31.6 ± 3.5 μl (p≤0.001, Figure 10D). There was a tendency towards an increase in the number of non-micturition contractions (pointed by arrows in Figure 9B) after intravesical VEGF, although the difference did not reach statistical significance (Figure 10E). Other cystometric parameters such as intermicturition pressure interval, threshold pressure and basal pressure were unaltered by VEGF treatment. Additional group of mice (N=5) underwent intravesical instillations with PBS and cystometric parameters were recorded at the same time points as in the VEGF group. Intravesical PBS did not significantly affect the function of the urinary bladder (Figure 10A-E). In addition, the baseline values did not differed between the PBS and VEGF groups and, therefore, baseline values were combined in Figure 10.

### Intravesical VEGF caused an up-regulation of voltage gated Na^+ ^channels (VGSC) in bladder DRG neurons

It is well established that bladder inflammation causes an increase in VGSC expressed in sensory neurons receiving input from the urinary bladder [21]. In this set of experiments we aimed to determine if intravesical VEGF would cause any changes in VGSC, thereby, affecting neuronal excitability of bladder projecting afferents.

**Figure 11:**
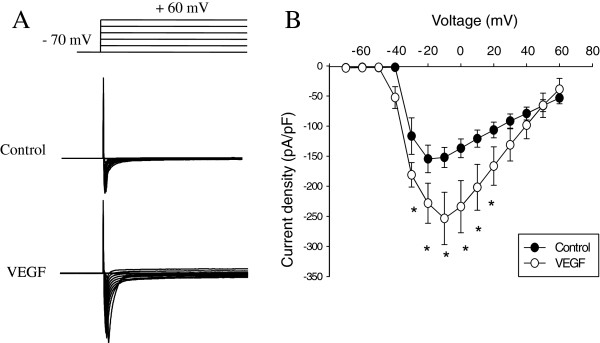
**The amplitude of voltage-gated Na**^**+ **^**currents is increased in bladder sensory neurons isolated from VEGF-treated mice. **A, Top panel presents the scheme of voltage protocol for recordings of voltage gated Na^+ ^currents including depolarizing pulses from -70 mV to +60 mV ( 10 mV increments) from the holding potential of -70 mV. Lower panel shows representative raw traces recorded from VEGF group 2 weeks after the beginning of the treatment. **B**, Current-voltage (I-V) relationship of the total Na^+ ^current recorded in bladder DRG neurons after intravesical application of VEGF (2 weeks). * - p≤0.05 when compared to control group.

Retrograde labeling of lumbosacral sensory neurons with Fast Blue allowed identification of bladder projecting DRG cells used for electrophysiological recordings and data analysis. Bladder inflammation caused by intravesical VEGF triggered an increase in the amplitude of total Na^+ ^current recorded from bladder afferent neurons. Representative raw recordings of total Na^+ ^current obtained from the control (intravesical PBS) and experimental (intravesical VEGF) groups are presented in Figure 11A. The current–voltage (I-V) relationship of total Na^+ ^current normalized to the cell size shows that these neurons produced a large amplitude Na^+ ^current upon membrane depolarization, reaching maximal amplitude at -20 mV (Figure 11B). VEGF application increased the peak amplitude of total Na^+^ current in bladder DRG neurons at -10 mV from -152.1 ± 16.8 pA/pF (n=11) in the control group to -253.5 ± 43.5 pA/pF (n=9) in the VEGF group (p≤0.05). Significant enhancement of total Na^+ ^current was observed at all voltages from -30 mV to +20 mV (Figure 11B).

**Figure 12:**
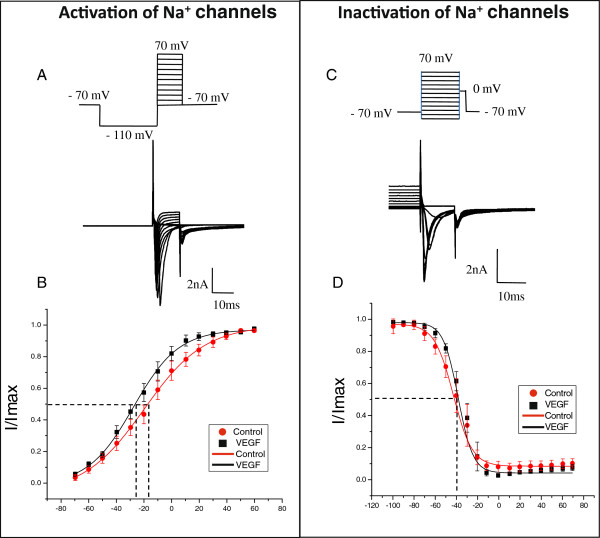
**Kinetics of voltage gated Na**^**+ **^**channels recorded in bladder DRG neurons. A**: The protocol (top panel) of steady-state activation and raw traces (bottom panel) of total Na^+ ^current. The steady-state activation of VGSC was assessed by using a three-pulse protocol with a negative pre-pulse to -110 mV and a series of short pulses of 10 ms duration from -110 mV to +70 mV to activate Na+ currents. **B**: Voltage dependence of steady-state activation in bladder neurons from control and VEGF treated animals. Please note a leftward shift in the group with VEGF instillations suggestive of channel opening at more negative potentials. **C**: The protocol of steady-state inactivation (top panel) and raw traces of the recorded Na^+ ^current (bottom panel). The amplitude of steady-state inactivation was measured at 0 mV after 150 ms depolarizing pulses ranging from -100 mV to 70 mV. **D**: Voltage dependence of steady-state inactivation of Na^+ ^channels in lumbosacral bladder DRG neurons was not different in the control group and VEGF-treated mice.

We next assessed the kinetic parameters of total Na^+ ^currents after the induction of neurogenic bladder inflammation caused by VEGF. The steady-state activation was studied by using a three-pulse protocol with a negative pre-pulse to -110 mV and a series of short depolarizing pulses (10 ms duration) to activate Na^+ ^currents (Figure 12A, top panel). The amplitude of steady-state activation was measured at the peak of tail current upon the voltage step to -70 mV, normalized and plotted as I/I_max _against the voltage (Figure 12A, lower panel). Intravesical VEGF led to the leftward shift in the steady-state activation of total Na^+ ^current by 8 mV (V_1/2_=-19.9± 2.6 mV in the control group *vs* -27.6± 2.0 mV at 2 weeks of treatment, Figure 12B, p≤0.05). The amplitude of steady-state inactivation was measured at a series of membrane depolarizing steps ranging from -100 mV to +70 mV (Figure 12C). Bladder inflammation did not affect the parameters of steady-state inactivation of total Na^+ ^current in bladder sensory neurons (Figure 12D).

### VEGF triggered an enhanced abdominal sensitivity to mechanical stimulation with von Frey filaments

**Figure 13:**
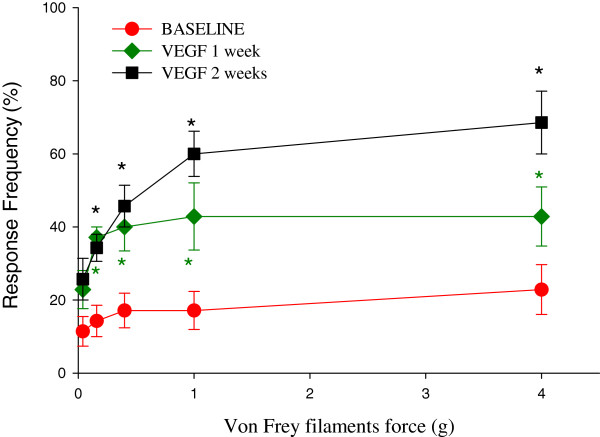
**VEGF triggered an enhanced abdominal sensitivity in response to mechanical stimulation with von Frey filaments.** Effects of intravesical VEGF on the development of viscerosomatic hyperalgesia as measured by the response to mechanical stimulation of the lower abdominal area using von Frey filaments. * - p≤0.05 when compared to the control group. This data suggests that intravesical VEGF induced the development of abdominal hypersensitivity in pelvic area which could be a reflection of abdominal discomfort/pain.

Abdominal sensitivity was tested in a separate group of mice (N=7) before and after bladder treatments with VEGF. Figure 13 summarizes the frequency of responses to von Frey filament testing in the lower abdominal area before, and 1 week and 2 weeks after intravesical instillations of VEGF in the same group of mice. The response frequency correlated with the applied force, reaching a plateau of 20% at the maximal tested force of 4 g (Figure 13). One week after VEGF treatment, mice became more sensitive to the filament testing and responses reached more than 40% at lower forces of 0.16 g and 0.4 g (Figure 13, p≤0.05 to baseline). Two weeks after the treatment the frequency response showed significant differences with stimuli of 1 and 4 g filaments in comparison to 1 week (Figure 13, p≤0.05 to baseline).

These results provide evidence that intravesical VEGF leads to an increased viscerosomatic response to cutaneous stimulation in the pelvic region. Such a response is usually associated with abdominal discomfort and/or pelvic pain.

## Discussion

The major findings of the present manuscript are: 1) the instillation of VEGF produces an increase in sensory nerve density, presumably by nerve sprouting, that reaches a maximum at 2 weeks and declines by 4 weeks; 2) in addition to significant increase in sensory nerve fibers, VEGF increases the density of bladder cholinergic nerve fibers; and 3) the increase in nerve density produced by VEGF results in altered bladder function and visceral sensitivity. The unique feature of our findings is that VEGF produces an increase in nerve density via urothelium.

The nervous and vascular systems share several anatomical parallels. Both systems utilize a complex branching network of neuronal cells or blood vessels reaching all regions of the body. The anatomical similarity of the nervous and vascular systems suggests that axons might guide blood vessels and vice-versa [22]. Indeed, signal molecules produced by peripheral neuronal cells, such as VEGF [12], guide blood vessels [14] and signals from vessels, such as the neurotrophins NGF and NT-3, are required for, and orchestrate extension of neurons adjacent to vessels [23]. In this manner, the neuronal and vascular systems are well organized and coordinated in normal adult tissues. However, in chronic inflammatory states particularly in the LUT, little is known about how the nerve-vessel relationship functions and whether it could underlie the chronic pain syndrome observed in patients with disorders of the lower urinary tract. In this context, this manuscript presents a body of evidence implicating VEGF signaling in the enhanced innervation of the urinary bladders in mice and the consequent alteration in mechanical responses and visceral sensitivity.

Interest in guidance molecules, and particularly VEGF, modulating both vascular and neuronal pathology is emerging [14]. Changes in VEGF levels are associated with alterations in the vascular system of the urinary bladder [17]. VEGF is increased in bladders of patients with painful bladder syndrome, and this increase is associated with glomerulations on hydrodistension [24]. However, increased bladder VEGF is not observed in patients who do not show petechial bleeding or in controls [24], suggesting that VEGF levels are associated with those PBS patients exhibiting alterations in the bladder microvascular system.

At this moment, it is not readily apparent which of the VEGF receptor subtypes mediates the bladder neuroplasticity in the mouse model. Both VEGFR1 and VEGFR2 as well as NRP1 and NRP2 are highly expressed in urothelium and intramural ganglia [25]. We also reported that control human bladders urothelium present a predominance of VEGFR1 and NRP2 over VEGFR2 and NRP1 immunoreactivity and that PBS patients present a decrease in VEGFR1 and NRP2 expression [26]. Nevertheless, our results strongly suggest a new and blossoming VEGF-driven processes in the bladder that may be a putative target in neuronal plasticity. However, the role of VEGF pathway in bladder neuroplasticity is in its infancy. In contrast, the roles of NGF and BNDF in neuroplasticity are well established in bladder pathology (e.g., due to spinal cord injury) and have resulted in the testing of NGF-/BNDF-antibodies (or siRNA knockdown) as possible therapeutic options. Therefore, it is tempting to propose that VEGF neutralizing antibodies, such as avastin, or VEGF receptor antagonists may be of benefit to reduce inflammation-induced bladder neuronal plasticity. However, it has to be kept in mind that this growth factor is necessary not only for developing vessels and angiogenesis but also VEGF signaling is required for vascular homeostasis [27] and the consequences of reduced levels of VEGF can impact the kidney vasculature as seen in pre- eclampsia [28]. A promising alternative for neutralization of VEGF seems to be the blockade of neuropilins by engineered antibodies [29]. However, it is too early to predict whether neutralization of neuropilins will have any deleterious effect on the established vasculature.

The rationale for the methodology employed here is based upon our previous observations that VEGF is taken up by the intact urothelium. We showed that following intravesical instillation of a fluorescent VEGF tracer (scVEGF/Cy5.5 ) which only internalizes in cells expressing active VEGF receptors [30], results in accumulation of this growth factor in suburothelial layers [31,32]. After binding these receptors, VEGF may be transcytosed by the urothelial cells into deeper suburothelial layers. Alternatively, VEGF could affect the permeability of the urothelium through mechanisms reminiscent of VEGF’s effects on vascular permeability, which results in paracellular transport of VEGF. Indeed, the protein constituents comprising this highly effective urothelial barrier (tight junctions), occludins [33], claudins [34], and zonula occludens-1 [35] have been recently studied in detail [36] and are known targets of VEGF-mediated effects on vascular permeability. After the uptake, VEGF produces both bladder inflammation and changes in neuronal plasticity [17]. The hypothesis that VEGF is taken up by the urothelium was substantiated by the following findings: 1- VEGF receptors are expressed in the mouse [32] and human bladders [31]; 2-VEGF neutralizing antibodies significantly reduced inflammation and neuronal plasticity induced by intravesical Bacillus Calmette-Guérin (BCG) stimulation [17]; 3- An antibody targeting neuropilins (VEGF co-receptors) reduces bladder inflammation [37]; 4- VEGF itself reproduced the findings obtained with BCG by causing bladder inflammation and sensory nerve plasticity [17].

### Increase in cholinergic fibers

Although an increase in sensory nerve density, particularly those expressing TRPV1-IR, has been proposed to underlie pain sensation and neurogenic detrusor overactivity [38], our past work did not explore whether the increased nerve density also resulted in altered function [17]. In order to investigate motor nerves, we used a unique mouse model expressing a fluorescent protein under the endogenous *Chat* gene promoter, we present evidence that direct application of VEGF into the mouse bladder increases the density of peripheral cholinergic nerves. To the best of our knowledge this increase in cholinergic nerve fibers represents a new finding that was only possible by the use this ChAT transgenic mouse, a model which will open a new area of research on the role of VEGF and its receptors in bladder motor function.

### How does VEGF produce its effects?

VEGF and its receptors are known neuronal guidance molecules and, therefore, it is expected that they affect nerves. However, the inflammation induced by VEGF may be another possible mechanism leading to an increase in neuronal density. Indeed, VEGF mediates inflammation in the bladder as shown by the findings that instillation of VEGF causes vasodilation, edema, and macrophage recruitment, hallmarks of inflammation [17], while application of neutralizing VEGF antibodies significantly reduce bladder inflammation [37]. At this time, there is no definitive evidence suggesting a specific inflammatory cell regulating bladder nerve plasticity. However, given the known trophic effects of VEGF on neurite growth prolonged survival of neurons [39,40], and reinnervation following local nerve damage [41,42], it is reasonable to propose that inflammatory cells producing VEGF may mediate these growth effects on neurons. This new appreciation of VEGF signaling in bladder inflammation is supported by emerging evidence that VEGF is increased at the site of inflammation, and that infiltrating lymphocytes and other inflammatory cells may represent additional sources of VEGF [43]. The involvement of cholinergic nerves on bladder inflammatory responses to VEGF suggests a cross-talk between the autonomic and immune systems. Whether the immune system is functionally and anatomically connected to the bladder nervous system remains to be determined. However, a recent investigation proposes that afferent and efferent signals transmitted in the vagus nerve modulate innate immune responses and are components of an inflammatory reflex [44,45]. Therefore, it is fair to propose that VEGF increases the cross-talk between the immune and autonomic systems.

### VEGF alters bladder function

We showed for the first time that VEGF affects bladder function and modulates micturition reflex pathways.

Analysis of urodynamic parameters recorded in conscious mice confirmed the suggested role of VEGF in modulation of micturition reflex pathways. It is known that exogenous VEGF (or hypoxia induced upregulation of the growth factor) can lead to detrusor and urothelial hypertrophy and hyperplasia [46]. In addition, previous immunohistochemical analyses of human specimens detected increased innervation in the suburothelial and detrusor layers of the urinary bladder in PBS patients [47,48]. In our mouse model, we established that intravesical VEGF treatment resulted in an increase in the density of ChAT fibers in both the detrusor smooth muscle and urothelial layers. This increase in nerve density was associated with altered bladder function as indicated by a decrease in the duration of intermicturition interval, reduced voiding pressure, micturition volume and bladder capacity during continuous filling cystometry. At this time, the individual contributions of sensory and motor nerves to the VEGF-induced increased bladder motility are not clear. Blockade of TRPV1 with capsazepine may shed some light in this respect.

Our results are consistent with other studies which established similar urodynamic changes in animal models of bladder irritation/inflammation [49-51]. For instance, overexpression of neurotrophic nerve growth factor (NGF), a well-known modulator of neural plasticity, in the urinary bladder of mice caused bladder hyperreflexia associated with increased voiding frequency [7,52]. These changes were accompanied by an increased density of calcitonin gene-related peptide, SP and neurofilament (NF) 100 positive fibers, as well as tyrosine hydroxylase-positive sympathetic nerve fibers within the suburothelial nerve plexus of the urinary bladder [7]. Additionally, expression of several TRP channels, including TRPA1, TRPV1, and TRPV4, was increased in the urinary bladder of mice over-expressing NGF [53]. Interestingly, the urinary bladder phenotype observed in mice with urothelial overexpression of NGF was associated predominantly with the afferent limb of the micturition reflex, whereas our results provide evidence that VEGF affects both afferent and efferent neural pathways. Our data confirmed the suggestion that VEGF may be a potent modulator of neural plasticity in the LUT. Other investigators also suggested that VEGF is a more potent stimulator of neuronal plasticity compared to a number of different neurothrophic factors [54-56]. However, a cross-talk between VEGF and neurotrophins cannot be discarded. On one hand administration of VEGF can support and enhance the growth of regenerating nerve fibers, probably through a combination of angiogenic, neurotrophic, and neuroprotective effects [57] and conversely, neurotrophins, such as NGF have been described as pro-angiogenic factors [58]. On the other hand VEGF had neurotrophic effects comparable with BDNF, NT3, or NT4 on the rat isolated pelvic ganglia in culture, [54]. In addition, VEGF was found to be more potent than BDNF in inducing ChAT-expressing fibers [54,55]. Moreover, the synergistic biological activity of VEGF and NGF [59] is supported by the finding that mechanical stretch of sympathetic neurons seems to induce VEGF expression via a NGF and CNTF signaling pathway [60]. An intriguing recent hypothesis explaining the cross-talk between VEGF and neurotrophins proposes the convergence of putative signaling downstream of receptor tyrosine kinases [61]. In this work, Kidins220 /ARMS (Ankyrin repeat-rich membrane spanning) was identified as a main player in the modulation of neurotrophin and VEGF signaling *in vivo*, and a primary determinant for neuronal and cardiovascular development [61]. In support of this hypothesis, it was demonstrated that Kidins220 interacts with neurotrophin, VEGF, ephrin, and glutamate receptors, and is a common downstream target of several trophic stimuli [61,62]. Adding to the cross-talk between neurotrophins and VEGF on neuronal plasticity, the present results go one step further by indicating that VEGF alters both sensory (TRPV1) as well as motor (ChAT) nerves. Our present results suggest the idea that across-talk between VEGF and neurothropins controls bladder motor (ChAT) nerve plasticity.

Electrophysiological recordings *in vitro* and *in vivo* revealed several distinct classes of afferent fibers that participate in transmission of sensory signaling upon physiological bladder filling, noxious distension, chemical irritation and inflammation [63]. Sensory neurons located within DRG are the first cells to receive afferent input from the pelvic viscera and, therefore, play a substantial role in the development of visceral sensitivity and pelvic discomfort during pathophysiological conditions. DRG neurons express several types of ion channels including TRPV1 and voltage-gated sodium channels (VGSC), both of which are well known transducers of nociceptive processing in pain pathways [64,65]. Experiments utilizing animal models of acute and chronic inflammation in the genitourinary tract showed an increased excitability of DRG neurons receiving direct input from the affected organs [49,66,67]. In this study, we determined that instillations of intravesical VEGF caused an up-regulation of VGSC in bladder sensory neurons identified by retrograde labeling. Overexpression of VGSC in bladder DRG cells is associated with increased neuronal excitability and enhanced firing rate [21]. Our results also support the data from human studies which suggested that abdominal pain and altered bladder and pelvic hypersensitivity in patients with OAB and PBS may involve organizational and/or functional changes in visceral afferent pathways when bladder sensory neurons become sensitized and hyper-responsive to normally innocuous stimuli such as bladder filling [68,69].

Multiple sodium channel isoforms are expressed in DRG neurons [70]. Sodium channels play a central role in neuronal electrogenesis, therefore, variations in the level of expression of any one of the sodium channel isoforms could, in principle, alter their level of excitability [71]. However, a number of modulatory factors such as neuronal functional status, homeostatic regulation of ion channel expression, post-translational modifications, and interactions with regulating molecules and trophic factors can also significantly affect neuronal excitability [70]. For instance, brief exposure to NGF, interferon gamma, epidermal growth factor or basic fibroblast growth factor can induce an up-regulation of expression of Na_V_1.7 channel [72]. Interactions of Na^+ ^channels with partner molecules including NGF [73,74], GDNF [73], contactin [75,76], annexin [77], gabapentin [78], and other modulators [79] were established to regulate expression of multiple sodium channel isoforms. Based on these observations, we suggest that the effects of VEGF treatment on Na^+ ^channels in our study could be associated either with the changes in the expression ratio between different Na^+ ^channel isoforms in bladder sensory neurons or modulation of Na^+ ^channel function by regulatory molecules as outlined above [70]. Additional studies are warranted to identify the exact mechanisms of VEGF action on specific Na^+ ^channels isoforms and electrical activity of bladder sensory neurons.

The results of behavioral experiments revealed, for the first time, that intravesical VEGF induced the development of abdominal hypersensitivity detected by mechanical stimulation of the lower pelvic region. These effects may be explained, in part, by the ability of VEGF to increase the density of SP and TRPV1 positive fibers [17]. This suggestion correlates with the previously published studies, which confirmed participation of TRPV1 in the development of abdominal hyperalgesia and neuropathic pain [80]. Results with TRPV1 knockout mice support the role of TRPV1 in mediating changes in sensitivity. Wang et al. determined that abdominal hyperreactivity and cutaneous allodynia were significantly diminished in these genetically modified animals although the lack of functional TRPV1 receptors did not improve the histological changes in the inflamed bladder induced by either cyclophosphamide (CYP) or acrolein [51]. In addition to the involvement of TRPV1 afferents in pelvic sensitivity, other receptors and molecules can also contribute to abdominal hyperalgesia depending on the model and nature of chosen inflammatory agents [81]. Thus, increased peripheral sensitivity in mice with bacterial cystitis was related to activation of toll-like receptor 4 [82]. In the acrolein model of bladder inflammation in rats, increased mechanical sensitivity was conveyed, in part, via NGF and trk receptors [83]. Likewise, inflammatory events experienced earlier in life were established to trigger long lasting changes in sensory pathways leading to altered pelvic sensations during the adulthood [84,85]. Altogether, our data provide direct evidence that VEGF-induced neurogenic inflammation in the urinary bladder is associated with significant structural and functional changes that may play a key role in the development of neurogenic bladder dysfunctions in humans.

## Conclusion

The discovery that neuronal guidance molecules such as neuropilins function as co-receptors for VEGF has opened up a new field of VEGF research and has even revealed potentially new roles for VEGF in axonal growth [86]. In other words, sprouting of neuronal axons and vessels appear to use common molecular mechanisms for navigation based upon NRP-VEGF interactions [87].

We previously provided strong evidence indicating that the mouse [25] and human urothelium [31] express an extraordinary level of VEGF receptors and that the expression of these receptors is fundamentally altered in bladder biopsies of PBS patients [31]. Additional results supported the hypothesis that the mouse bladder urothelium actively internalizes VEGF [31].

Nevertheless, the function exerted by VEGF in the LUT is not clear. In both neuronal and vascular cells VEGF is known to increase permeability, prevent apoptosis [88], and promote cell survival [89] and proliferation [90]. Whether VEGF exhibits the same functions on urothelial cells remains to be determined. The results presented in this manuscript indicate that VEGF also participates in bladder neuronal plasticity and that the increased nerve density is accompanied by alterations in bladder function and visceral sensitivity.

## Methods

### Animals and experimental groups

All animal experimentation conformed to the APS’s Guiding Principles in the Care and Use of Animals and was approved by the OUHSC Animal Care & Use Committee (protocol #08-105), University of Wisconsin-Madison (protocol M02497), and University of Pennsylvania School of Medicine (protocol # 802979)

For the visualization of cholinergic neurons we used genetically engineered mice with an "IRES-Cre" sequence inserted downstream of the stop codon such that *cre* expression is controlled by the endogenous *Chat* gene promoter (B6;129S6-*Chat*^*tm1(cre)Lowl*^/J.; Jackson Laboratories stock # 006410). We mated the ChAT-cre mice with Rosa stop *td* tomato mice. The resulting offspring express the fluorescent protein (td tomato) in all cholinergic cells and fibers, as the cre deletes the stop signals flanking the td tomato sequence. In these mice, *Chat* gene expression, however, is unaffected. For simplicity, this mouse strain will be referred as ChAT mice.

Adult C57BL/6J and ChAT female mice (10-12 wks old, Jackson Laboratory) were used in this study and maintained on a 12-hour light/dark cycle with *ad libitum* access to water and food.

Intravesical instillation was described in [91]. Briefly, 2-4 month-old female mice were anesthetized with isoflurane (between 1 and 2.5% titrated to effect) and transurethrally catheterized with a polypropylene catheter (24 gauge; ¾ in.; 19 mm long, inside diameter 0.47 mm, outside diameter 0.67 mm; Angiocath, Becton-Dickinson, Sandy, UT). Test compounds were instilled via a syringe attached to the catheter at a slow rate to avoid trauma and vesicoureteral reflux. 100 μl of one of the following substances were instilled: pyrogen-free saline (PBS; controls) or mouse recombinant VEGF, also known as VEGF-A or VEGF_165 _(6.41 nM in 100 μl; ProSpec-Tany TechnoGene Ltd [Rehovot 76124, Israel; catalog # MGC70609]). To ensure consistent contact of substances with the bladder and to avoid reflux or leakage, the catheter was occluded and left in place for 30 minutes. Mice received one or twice weekly innstillation of VEGF, as described in the particular figure legend. The groups of mice were euthanized one week post-instillation, and the urinary bladders were removed and examined as whole mounts. Subsequently, the urinary bladders were sectioned and visualized as cross-sections and immunofluorescence was used for image analysis of cholinergic and sensory nerves.

### Functional Studies

C57BL/6J female mice were randomly divided into three experimental groups: 1 – cystometry studies (N=5); 2 - control group for electrophysiological experiments (intravesical PBS, N=5); 3 – VEGF group (intravesical VEGF, N=5). Animals in all groups first underwent survival surgeries as described below followed by 3 intravesical instillations of either PBS or vascular endothelial growth factor (VEGF, 100 μL, 6.41 nM in 100 μl, groups 1 and 3) within a 2-week period (every 4^th^ day), see Table 1.

**Table 1:** Experimental scheme for functional studies

**DAYS**	**0**	**1**	**5**	**7**	**9**	**14**
PROCEDURES						
		1^st^ instillation of VEGF	2^nd^ instillation of VEGF		3^rd ^instillation of VEGF	
	Cystometry (Baseline)			Cystometry (1 week)		Cystometry (2 weeks)
				Von Frey Testing (1 week)		Von Frey testing (2 weeks)
						Electrophysiology (2 weeks)

### Bladder whole mounts

Urinary bladders were isolated from ChAT mice, the urine was drained, and tissues were fixed in 4% paraformaldehyde for 4-6 hours at room temperature or overnight at 4°C. After fixation, the tissue was washed in PBS twice and placed in 1% triton X-100 for 4-6 hours at room temperature or overnight at 4°C. After the tissue was washed in PBS, the primary antibodies were added for 4-6 hours at room temperature or overnight at 4°C, washed off in PBS, and the secondary antibodies were added for 4-6 hours at room temperature or overnight at 4°C. Whole mounts were visualized and photographed with a dissecting fluorescence microscope (Nikon SMZ 1500 microscope equipped with high-resolution Plan 1.6 WD24 objective lenses). For detailed visualization of ChAT and TRPV1 nerves along with blood vessels, some of the whole mounts were blunt dissected to separate the urothelium together with the suburothelium/lamina propria [92] from the detrusor smooth muscle. Photomicrographs were taken with the lamina propria facing upward and the urothelium downward or the detrusor smooth muscle facing upward and the adventitia downward. Subsequently, tissues were frozen in 1:1 tissue freezing media (TFM, Triangle Biomed Sciences) and OCT (Tissue Tek®) and sectioned with a cryostat. Cross-sections were examined by immunofluorescence, as described below.

### Immunofluorescence (IF) of mouse tissues

Urinary bladders were processed for IF according to published methods [93]. For all tissues, appropriate cross-sectional morphology was confirmed by H&E staining and examination by light microscopy prior to preparing slides for IF labeling. Frozen sections (10, 15, or 40 μm thick, as indicated in the figure legend) were post-fixed in 1% MeOH-free formaldehyde for all stains. Briefly, slides were blocked for 35 minutes with 5% normal donkey serum (NDS; Jackson Immunolabs), then co-incubated with primary antibodies in 0.5% NDS for 90 minutes in a humidified chamber or overnight at 4°C. When double IF was used, following brief rinses with PBS, slides were co-incubated with both secondary antibodies at the same time. Controls included slides labeled only with individual primary or secondary antibodies.

### Primary antibodies

TRPV1 antibody (1:10,000) was raised in rabbits against the 15 C-terminal amino acids of the rat TRPV1 sequence [94]. Commercially available antibodies included: rabbit anti-human protein gene product 9.5 [PGP9.5] (Neuromics; catalog # RB12103, 1:1500 dilution), rabbit anti-mouse substance P (Millipore; catalog AB1566; 1:250 dilution), guinea pig anti-rat substance P (Millipore; catalog AB15810; 1:1000 dilution), rat anti-mouse-derived endothelioma cell line [CD31] (BD Pharmingen; #550274; 1:200), rabbit anti-β III tubulin [TuJ1] (Gift from Dr. Anthony Frankfurter; 1:250).

### Secondary antibodies

All secondary antibodies were used at a 1:500 dilution and included donkey anti-rabbit IgG Alexafluor 488 and 546 conjugate (Molecular Probes), donkey anti-goat IgG Alexafluor 546, donkey anti-rat IgG Alexafluor 488, goat anti-guinea pig 546 ,donkey anti-rat Cy5 (Jackson ImmunoResearch), donkey anti-rabbit Dylight 488), donkey anti-rabbit Cy5. Slides were washed, counterstained with 4’, 6-diamidino-2-phenylindole (DAPI), and coverslipped.

### Image analysis

A Nikon A1R scanning confocal microscope (Melville, NY) controlled by NIS-Elements C (Nikon) was used to image whole mounts and cross sections at UW-Madison. For image analysis, all tissue cross-sections were viewed with a Nikon Eclipse TE 2000-S inverted fluorescent microscope at OUHSC and imaged at room temperature using a digital CCD camera (Roper Scientific; Sarasota, Florida 34240) driven by NIS-Elements AR 3.0 Imaging software. A control slide stained only with secondary antibody was used to determine exposure time and to set minimum background fluorescence levels for each fluorophore imaged. Once set, exposure times were not changed during acquisition of each respective fluorophore in the staining series. Staining was considered positive only when the acquired signal exceeded the established background. Absence of signal bleed-through was determined using previously optimized multi-acquisition settings on single fluorophore stained slides. DAPI staining was viewed using a DAPI filter set (340-380nm ex, 435-485nm em). Imaging of Alexafluor 488 utilized an excitation filter of 465-495nm and an emission filter of 515-555nm. Alexafluor 546 was imaged with an excitation of 528-553nm and 590-650nm emission range.

### Quantification of nerve fibers

Histologically nerve fibers undulate in and out of the plane of the section, sometimes appearing as linear structures, and sometimes as punctate staining, indicating presumed nerves in cross-section. Therefore, the following parameters were used in order to exclude structures above or below a certain size as being potentially non-neuronal and to exclude inflammatory cells since monocytes and macrophages have been reported to express TRPV1 [95]. In this context, for image analysis of nerves, the NIS-Elements AR 3.0 Imaging software was set to count only structures with length between 0.19-500 μm and width 0.19-2.5 μm. [Length is a derived feature appropriate for elongated or thin structures. Length = (Perimeter + sqrt (Perimeter^2^- 16*Area))/4]; and Width is a derived feature appropriate for elongated or thin structures. It is based on the rod model and is calculated according to the following formula: Width = Area/Length].

To meet the independent randomized sampling assumption required for our statistical test(s), the following measures were taken: blinding the reader to treatment groups and picking a random starting position and proceeding clockwise with 6-10 non-overlapping images. As 12-20 fields are necessary to view the whole bladder cross-section at 400X magnification, the sampling of 6-10 non-overlapping images represented half of the entire bladder cross-section. The area occupied by cells stained positively with td tomato or TRPV1 antibody was calculated as percent of the total area of the region of interest (ROI), as indicated in the individual figure legend and the results of 6-10 fields were averaged. This procedure was repeated for all bladders used per treatment group and the results are expressed as mean ± SEM of cross-sections. The data were examined to determine if the distributions were homoscedastic and Gaussian. As these conditions were met, groups were compared through a two-sample Student’s t test. An alpha of 0.05 was considered statistically significant. *P*-values were adjusted for multiple comparisons through a Bonferroni correction.

### Surgical procedure to catheterize the urinary bladder in mice

Mice included in the group for urodynamic evaluation of the urinary bladder function (awake cystometry) underwent the following survival surgical procedure to insert bladder catheters. An animal was anesthetized with isoflurane (VEDCO, St. Joseph, MO), and a PTF catheter with a blunted end (Catamount Research, St. Albans, VT) was sutured in place at the bladder dome and tunneled out the abdomen to the nape of the neck where it was then inserted into the end of a 22-gauge angiocath iv catheter. Upon determination of the optimal length, the PTF catheter was affixed to the angiocath with super glue. The angiocath was first tested with a gentle saline infusion to reveal no leak at the bladder, and then capped and the abdomen was closed in layers. The angiocath was anchored to the fascia and skin of the neck using two to three 3–0 Vicryl sutures. Animals were kept in individual cages to avoid possible damage to the catheters by their cage mates. Mice were allowed to recover from surgery for 4 days followed by cystometric evaluation of bladder function under normal conditions (baseline cystometry). After initial urodynamic evaluation mice received 3 intravesical instillations of VEGF as described above.

### Surgical procedure for labeling urinary bladder DRG neurons

Mice were anaesthetized with 2% isoflurane and held on a warming pad inside a fume hood to minimize the investigator`s exposure to the anesthetic. A midline laparotomy was performed under sterile conditions to gain access to the urinary bladder. Urinary bladder was exposed and Fast Blue (Polysciences Inc., Warrington, PA, USA; 1.5% w/v in water) was injected into the urinary bladder wall (detrusor at 6 – 10 sites) using a Hamilton syringe with 26-gauge needle. The total volume of dye injected into the bladder was 20–25 μl. Adjacent pelvic organs were isolated with gauze to soak up any spills and prevent the labeling of adjacent structures during injections. Additionally, the needle was kept in place for 30 minutes after each injection. Any leaked dye was removed with a cotton swab before placing the organ into the pelvic cavity. Incisions were sutured in layers under sterile conditions followed by subcutaneous injection of buprenorphine (0.5 mg/kg). Animals were allowed to recover on a warm blanket until they gained full consciousness and then were returned to their cages. Mice started treatments with intravesical VEGF (or PBS in the control group) 10 days after labeling of bladder sensory neurons.

### Urodynamic evaluation of bladder function

Conscious mice were placed in cystometry cages (16 cm width, 12 cm height, and 24 cm length) without any restraint and allowed to acclimate for 30 min. The tip of the exteriorized bladder catheter located at the base of the mouse neck was connected to a pressure transducer and an infusion pump of the cystometry station (Small Animal Laboratory Cystometry, Catamount Research and Development, St. Albans, VT) using a T-shaped valve. Room temperature saline solution (0.9% NaCl) was infused into the bladder at a rate of 10 μl/min. Voided urine was collected in the tray connected to a force displacement transducer integrated into the data acquisition system. Each animal was observed for up to six-eight voiding cycles. Urodynamic values were recorded continuously using data acquisition software (Small Animal Laboratory Cystometry, Catamount Research and Development). The following cystometric parameters were recorded and analyzed in this study: bladder capacity, pressure at the start of micturition, micturition rate, continuous intravesicular pressure, inter-micturition interval, and the number of non-micturition contractions. Non-micturition contractions were defined as increased values in detrusor pressure from the baseline that had amplitudes of at least a third of maximal pressure at the start of micturition. Each animal underwent baseline cystometric evaluation followed by intravesical instillations of VEGF. The second cystometric assessment was done after two VEGF instillations (1 week after the first dose) and the third cystometry was performed 3 days after completion of all three VEGF treatments at 2 weeks after the baseline measurement (day 0), Table 1.

Cystometric parameters were uploaded from the acquisition software into analysis software (SOF-552 Cystometry Data Analysis, Version 1.4, Catamount Research and Development Inc., St. Albans, Vermont). Maximum pressure at micturition, bladder capacity, micturition volume, number of non-micturition contractions, intermicturition interval and micturition rate indices were calculated. All data are expressed as the mean ± standard error of the mean (S.E.M). The results were statistically analyzed using one-way repeated measures ANOVA between baseline and 2 urodynamic assessments followed by Bonferroni`s post test, as appropriate (Systat Software Inc., San Jose, CA). A difference in values between the baseline and treatments was considered statistically significant at p≤0.05.

### Isolation of bladder DRG neurons for patch-clamp experiments

Animals were euthanized by overdose of sodium pentobarbital (130 mg/kg) 2 weeks after the beginning of VEGF treatments. Dorsal root ganglia were dissected and removed bilaterally at L6-S2 levels. The ganglia were treated with collagenase (Worthington, type 2, Biochemical Corp., Lakewood, NJ, USA) in F-12 medium (Invitrogen, Carlsbad, CA, USA) for 90 min in an incubator with 95% O_2 _and 5% CO_2 _at 37° C. Isolated ganglia were then rinsed in phosphate-buffered saline (PBS) and incubated for 15 min in the presence of trypsin (Sigma, St Louis, MO, USA; 1 mg/ml) at room temperature. The enzymatic reaction was terminated in Dulbecco’s Modified Eagle’s Medium (DMEM) containing 10% of fetal bovine serum (FBS). Single neurons were obtained by gentle trituration using fire-polished Pasteur pipettes in DMEM with trypsin inhibitor (Sigma; 2 mg/ml) and deoxyribonuclease (Sigma; DNase 1 mg/ml). The cell suspension was centrifuged for 10 min at 700 rpm (4° C), and supernatant was discarded. The pellet, containing sensory neurons, was resuspended in 2 ml of DMEM containing 10% FBS. Neurons were plated on poly-L-ornithine coated 35 mm Petri dishes. Isolated cells were maintained overnight in an incubator at 37° C with 95% O_2_/5% CO_2 _and were used for electrophysiological experiments within 24 hours.

### Electrophysiological recordings of voltage gated Na^+ ^currents from bladder dorsal root ganglion neurons

Bladder labeled neurons were identified with an inverted fluorescent microscope (Ti E2000–5, Nikon containing a specific filter for Fast Blue (UV-2A, Nikon). Only neurons exhibiting bright blue fluorescence (Fast Blue labeled) were used for Na^+ ^current recordings using the whole-cell patch clamp technique. For voltage clamp experiments the external solution contained (in mM): NaCl 45, TEA Chloride 30, Choline Chloride 60, KCl 5.4, MgCl_2 _1, CaCl_2 _1, HEPES 5, D-glucose 5.5, adjusted with NaOH to pH 7.4. Pipette solution for these experiments consisted of (in mM): L-aspartic acid 100, CsCl 30, MgCl_2 _2, Na-ATP 5, EGTA 5, HEPES 5 adjusted with CsOH to pH 7.2. CdCl (100 μM) was added to the external solution in order to block voltage gated calcium currents. Freshly made Amphotericin B (0.24 mg/ml, ACROS, NJ, USA) was added to the pipette solution for perforated whole cell recordings. Microelectrodes were fabricated from borosilicate capillary glass (Sutter instruments, Novato, CA) and had resistances of 2–5 MΩ when filled with internal solution. Recordings commenced 5 min after the establishment of whole cell access. Series resistance was compensated ≥80–85%, and the calculated junction potential was around 5 mV. Cells were excluded from analysis if uncompensated series resistance resulted in a maximum voltage error >5 mV or if the seal or access resistance were unstable. Recordings and analysis of kinetic parameters of voltage gated Na^+ ^channels were performed using previously established protocols [9].

pCLAMP software (Axon Instruments, CA) was used for data acquisition and analysis. All data are expressed as means ± SEM. Statistical significance between the groups was assessed by one-way ANOVA followed by Bonferroni`s test. Data with p≤0.05 were considered statistically significant.

### Assessment of visceral sensitivity using von Frey filaments

Mice were tested before and after intravesical instillations of VEGF. Irritation and/or inflammation in the pelvic viscera are associated with enhanced abdominal sensitivity due to convergence of visceral and somatic inputs on the second order neurons in the dorsal horn of the spinal cord [21]. This phenomenon is known as viscerosomatic (called hyperalgesia) and is measured by using mechanical stimulation with von Frey filaments on the lower abdominal/pelvic area. Mice were tested in individual Plexiglas chambers (6 x 10 x 12 cm) with a stainless steel wire grid floor (mouse acclimation period was 30 min before testing). Frequency of withdrawal responses was tested using five individual fibers with forces of 0.04, 0.16, 0.4, 1, and 4 g (Stoelting). Each filament was applied for 1–2 s with an interstimulus interval of 5 s for a total of 10 times, and the hairs were tested in ascending order of force. Stimulation was confined to the lower abdominal area in the general vicinity of the bladder, and care was taken to stimulate different areas within this region to avoid desensitization or "wind up" effects. Three types of behavior were considered as positive responses to filament stimulation: *1*) sharp retraction of the abdomen, *2*) immediate licking or scratching of the area of filament stimulation, or *3*) jumping as previously described [96].

## Authors’ contributions

This manuscript reports the results of studies developed at the University of Pennsylvania (UP), UW-Madison, and OUHSC. At the UW-Madison, under the directions of MLE, MRS anesthetized and instilled the ChAT mice and CSE developed the analysis of bladder whole mounts and immunofluorescence of mouse tissues. At the OUHSC, under the direction of MRS, CAD developed image analysis and quantification of nerve fibers. At the UP, under the direction of APM, QL developed the functional studies, including urodynamic evaluation of bladder function, isolation of bladder DRG neurons for patch-clamp experiments, electrophysiological recordings of voltage gated Na^+ ^currents from bladder dorsal root ganglion neurons, and assessment of visceral sensitivity using von Frey filaments. RS conceived the study, analyzed the results, and prepared the manuscript. All authors read and approved the final manuscript.
